# Do sheep-grazed pastures support insectivorous bat activity and bat species richness?

**DOI:** 10.1371/journal.pone.0341356

**Published:** 2026-01-23

**Authors:** Florian Wiesinger, Marcela Suarez-Rubio

**Affiliations:** Institute of Zoology, Department of Ecosystem Management, Climate and Biodiversity, BOKU University, Vienna, Austria; Institute of Geographic Sciences and Natural Resources Research Chinese Academy of Sciences, CHINA

## Abstract

Arable agriculture is usually associated with monoculture and the usage of pesticides, which jeopardize biodiversity and ecosystem processes. Grazing livestock can potentially benefit bats, however, most studies evaluate cattle, and it is unclear whether the results apply to grazing sheep. We assessed the effects of grazing sheep on insectivorous bat activity and species richness in southern Burgenland, Austria. We conducted acoustic surveys during the summer of 2019 on 49 pastures, which were in each case temporally divided into three categories (pre-grazed, grazed, and post-grazed). During the surveys, 20 of Burgenland’s 24 different bat species were detected. The most prevalent species was *Pipistrellus pygmaeus*. Small aerial insectivores showed significantly higher activity in grazed and post-grazed pastures compared to pre-grazed pastures, with no difference between grazed and post-grazed conditions. Frequent species (those occurring at ≥30% of sampled pastures) were more active in grazed than pre-grazed pastures but showed similar activity between pre-grazed and post-grazed pastures. At the species level, *Nyctalus noctula* activity was significantly higher in post-grazed compared to pre-grazed pastures. Overall bat activity, infrequent species activity, and species richness did not differ among grazing phases. Our results capture immediate responses during grazing and brief post-grazing resource pulses within days. Thus, comparisons with cattle should be cautious, as many cattle studies span weeks to months on larger, higher-biomass pastures. Apparent differences likely reflect timeframe, pasture size, stocking rate, and dung processes as much as livestock type, highlighting the need to consider livestock and management context when assessing bat responses and designing conservation-oriented grazing strategies.

## Introduction

Agriculture, a predominant form of land use worldwide, has profoundly transformed natural landscapes [1–3]. Arable agriculture is often associated with monoculture and the use of pesticides, which can jeopardize biodiversity and ecosystem processes [3,4]. Arable agriculture can have negative impacts on habitat quality and nutrient cycles, leading to biodiversity loss and changes in species assemblages [5–8]. Hence, arable agriculture, in particular industrialized agriculture, can be considered one of the main drivers of declining biodiversity in Europe [9–11]. Today, around 43% of Europe’s surface is agricultural land [12], reflecting the ongoing transformation of natural habitats into productive farmland [13]. While the effects of arable agriculture are well documented [e.g., 14], less attention has been paid to the ecological consequences of other agricultural practices, such as grazing livestock [13].

Domestic grazing livestock, including cattle, sheep, or horses, are descendants of herbivores that are now extinct or locally extirpated [15]. For example, cattle descend from the aurochs (*Bos primigenius*), sheep from the wild mouflon (*Ovis orientalis*), and horses from wild horses (*Equus ferus*) [16–18]. Historically, these wild herbivores played a key role in shaping the semi-open landscapes that were widespread across much of Central Europe during the late Pleistocene to early Holocene [16,19]. These semi-open landscapes were characterized by a mosaic of different habitats, such as forests and open meadows, which led to various biological niches and high biodiversity [9,20]. However, agricultural intensification has led to the decline of such semi-natural habitats, which are now restricted to specific areas and are highly vulnerable [21]. Where they persist, these semi-open landscapes play a crucial role in supporting biodiversity [22].

Grazing livestock can have various impacts on both biodiversity and ecosystem processes [23–25] depending on the grazing intensity [26,27]. Intensive grazing is characterized by high stocking density, grazing pressure, and foraging utilization [28] (i.e., the proportion of available plant biomass that is consumed by grazing livestock [29]). It can disturb soils (e.g., via soil compaction or altering nutrient cycling via animal dung [30]), which affects plant productivity [31–33]. Besides directly affecting plant diversity [34,35], it also affects the diversity of above-ground and below-ground organisms such as insects and soil animals [36], and increases the transmission of diseases from domestic to wild animals [37,38]. In contrast, extensive grazing is characterized by low grazing intensity and is associated with management practices, such as low external inputs (i.e., little or no fertilizer application), and infrequent mowing [39]. This approach offers multiple ecological benefits: protects soil from compaction and erosion [40]; promotes habitat heterogeneity by generating a mosaic of bare ground, taller and shorter vegetation, offering a range of microhabitats [41]; supports greater biodiversity, including insects that thrive under grazing compared with mowing [9]; and enriches soil fertility through deposition of livestock dung [27]. Many studies have shown that the richness of both animal and plant species is higher on extensively grazed pastures than on comparable intensively grazed, mowed, or wild areas [e.g., 9,42]. This can be attributed to the continuous and selective foraging of (different) livestock species, which creates a mosaic of ecological niches, enhancing biodiversity [9,43]. As a result of these positive impacts, extensive grazing is now widely used to manage protected areas due to its ability to enhance biodiversity and create structurally diverse landscapes [9,44,45].

Despite evidence that extensive grazing enhances biodiversity and promotes habitat heterogeneity, its potential to support insectivorous bats remains insufficiently understood. Bats, which are among the most threatened vertebrates in Europe, are protected under Annex II and IV of the Habitats Directive of the European Union [46]. These nocturnal mammals provide key ecosystem services, including pollination, pest control, and seed dispersal [47,48]. Studies suggest that extensive grazing can have positive effects on bats by creating a structurally diverse landscape that supports rich insect communities, which serve as prey for insectivorous bats [6,49–51]. For example, Reisinger and Schmidtmann [52] observed that two years after starting a project with extensively-grazing cattle and horses, the number of detected bat species increased from 5 to 11, with a corresponding rise in bat activity. Similarly, a nursery roost of *Rhinolophus ferrumequinum* in Germany increased from 37 adult bats in 2003 to nearly 200 in 2013 [5]. This population growth has been attributed to both the improvement in habitat structure and the establishment of extensively-grazing cattle within the bats’ hunting areas.

Extensive grazing can also enhance insect abundance by providing nutrient-rich dung, a resource that supports many insect species consumed by bats. For example, increases in dung beetle populations, resulting from grazing, have been linked to population growth in *R. ferrumequinum* bat colonies [53]. Additionally, extensive grazing prevents dense vegetation overgrowth and clutter, maintaining the open spaces required by many bat species [54]. At the same time, the presence of livestock attracts a variety of insects, including mosquitoes (Culicidae) and biting midges (Ceratopogonidae), which can carry diseases, such as the bluetongue disease or the Schmallenberg disease [55,56]. Many small bat species (e.g., *Pipistrellus* *sp.*) are key predators of such pests and may play an important role in controlling them [49]. This pest control function is expected to become increasingly important, as global warming facilitates the arrival of insect species carrying potentially dangerous diseases into temperate latitudes [55].

Although extensive grazing influences vegetation structure and insect communities, which in turn affects insectivorous bats, the specific effects of different grazing phases (i.e., pre-grazing, during-grazing, and post-grazing) remain little understood. During pre-grazing periods, vegetation tends to be taller and denser, potentially reducing the detectability or accessibility of insects for bats that forage near the ground or within clutter [42]. During grazing periods, both the presence of livestock and fresh dung create immediate disturbance and abundant insect prey, such as dung beetles and other insects that are attracted to both animals and their excrement [57]. During post-grazing periods, livestock are absent, but dung deposits remain, sustaining insect populations, albeit potentially with different composition or abundance than during active grazing [58]. Additionally, vegetation recovery during these periods leads to structural changes in the habitat, such as the regrowth of grasses and herbs, which can increase habitat heterogeneity [59]. This heterogeneity could provide a variety of foraging opportunities for bats, thereby sustaining diverse bat assemblages [60]. While studies have found significantly higher bat activity in grazed areas compared to ungrazed areas [49,51], few have directly compared bat activity and species richness across these temporal grazing phases, limiting our understanding of how short-term temporal changes in grazing management shape bat communities [61].

Most studies examining the influence of grazing livestock on bats have focused on cattle grazing systems [e.g., 9,49], which differ from sheep grazing systems in both grazing patterns and pasture management. Unlike cattle, which typically graze continuously over large areas, sheep are often managed in smaller pastures using rotational grazing practices. These involve moving sheep between paddocks three to four times during the grazing season (i.e., from late March to late December (personal communication with farmers). This rotational practice results in a more concentrated and clustered grazing impact compared to the relatively gentle and continuous grazing by cattle [9]. Due to these differences in livestock behavior and pasture management, it remains unclear whether findings from cattle grazing systems can be extrapolated to sheep grazing contexts.

Here, we evaluated bat activity and species richness across sheep pastures by comparing different grazing phases: pre-grazing, grazing, and post-grazing. Besides considering overall bat activity, we also evaluated the activity of small aerial insectivores (primary *Pipistrellus* spp. which are characterized by their small size and preference for feeding on small, flying insects (e.g., midges and other Diptera), frequent species (those occurring at ≥ 30% of sampled pastures), and infrequent species (those occurring at < 30% of sampled pastures). We hypothesized that bat activity and species richness would be higher on pastures with actively grazing sheep compared to those without sheep (pre-grazed, post-grazed) because active grazing creates ideal conditions—abundant insect prey [9,49,62] and suitable habitat structure [9,45,49,51,63]— that are lacking during pre-grazing (dense vegetation, fewer insects) and post-grazing (absence of disturbance despite residual dung). We also hypothesized that in pastures with actively grazing sheep, the activity of small aerial insectivores (e.g., *Pipistrellus pipistrellus*) would be higher because livestock attracts mosquitoes and biting midges, the preferred prey of these bat species [51]. On the contrary, the activity of species like *Myotis myotis* would be higher in post-grazed pastures, as they benefit from short vegetation that facilitates ground hunting for prey, such as dung beetles [49,64]. Other species that may exhibit similar behavior include other *Myotis* species, such as *Myotis nattereri*, which also forage close to the ground [65], as well as gleaning species like *Plecotus auritus*, which hunt insects from surfaces [66]. By investigating bat activity and species richness across different grazing phases, we aim to provide management suggestions for effectively protecting bats in extensive pastures with grazing sheep.

## Materials and methods

### Study area

**Fig 1:**
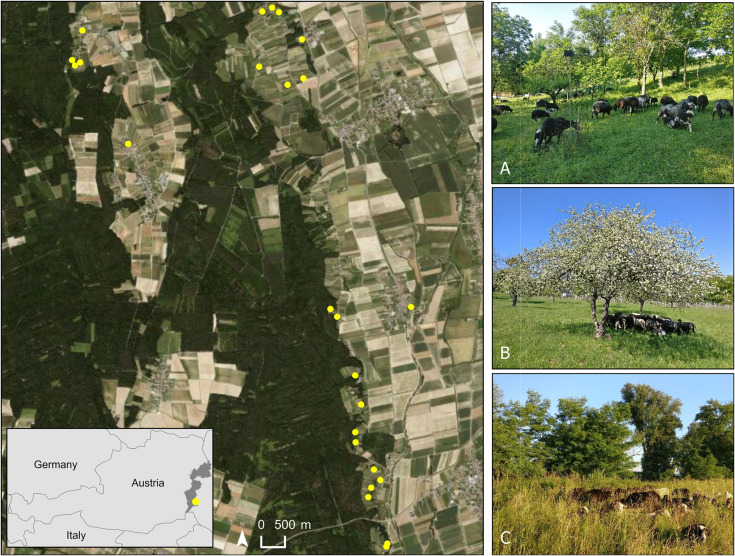
Location of the study area near the nature park ‘Weinidylle’ in Southern Burgenland, Austria. Sampled pastures are depicted as yellow points. Photos on the right are examples of three pastures: (A) former orchard, (B) dry meadow, (C) wet meadow (Sources World Imagery (basemap): Esri, Earthstar Geographics, and the GIS User Community; country boundaries: geoBoundaries [73]; photos Florian Wiesinger).

The study was conducted near the nature park “Weinidylle” in southern Burgenland, Austria (Fig 1). The nature park was founded in 1978, occupies an area of 7,270 ha, and is part of the European nature reserve “South Burgenland – hilly and terrace country”. The focus of the nature park is tourism, recreation, and education rather than conservation [67]. Habitats of the nature park include wet meadows, marshlands, vineyards, orchards, and dry grasslands. Vulnerable bat species for Europe, such as *Barbastella barbastellus* [68], *Myotis bechsteinii* [69], and *Myotis blythii* [70]*,* all included in Annex II of the Habitats Directive of the European Union [46], have been reported in the area [71]. The study area is situated within the Pannonian and Sub-Illyrian ecological regions, which are characterized by a mean annual temperature of 10°C and a mean annual precipitation of 700 mm. With less than 300 m a.s.l. the study area falls within the lowland altitude zone [72].

We sampled 49 pastures, which range from 0.2 to 1.5 ha, with a mean size of 0.4 ha (Fig 1). The pastures included orchards, former vineyards, dry meadows, and wet meadows. There were at least some trees in every pasture, but no natural water bodies. Three different flocks of sheep, all from the same breed (“Krainer Steinschaf”), with around 70 sheep each, rotated between pastures throughout the grazing season. During each sampling round, we included pastures representing all three grazing phases (pre-grazing, grazing, and post-grazing), ensuring that each grazing phase was represented in every sampling round within the same area and period. This design reduced potential interference between neighboring pastures. The distances between pastures, measured between their nearest edges, range from 68.4 m to 8.7 km, with a mean distance of 633.4 m. The average duration of a flock on each pasture was one week, depending on the vegetation height. Most of the sampled areas have been used as sheep pastures for the last 17 years. We received a permit to access and sample the pastures from Bioschafhof Elpons (Alexander Elpons).

### Bat surveys and call analysis

We used ultrasound recorders (Batcorder 1.0 and 2.0, ecoObs, Nuremberg, Germany) to survey the 49 pastures from May to September 2019. Each pasture was temporally divided into three categories: pre-grazed, grazed, and post-grazed. On each pasture, we placed one Batcorder for one night when no sheep were present in the pasture (pre-grazed), one night during the middle of a seven-day grazing period (grazed), and one night after the flock had left the pasture (post-grazed). On average, the interval between pre-grazed and grazed recordings was six days, and between grazed and post-grazed recordings, four days. In total, we recorded three nights per flock per pasture, for a total of 147 survey nights. The Batcorders were placed in the middle of each pasture, and the position did not change from pre-grazed, grazed, and post-grazed.

We deployed between two and three Batcorders simultaneously (i.e., on the same night) in sites close by to exclude any effect of temporal variation in local activity levels. Batcorders were installed under good weather conditions (i.e., no rain, temperatures above 10°C, Beaufort wind force < 3) on a 2 m-high pole and 2 m apart from any structure, like bushes and trees, to reduce echo recordings, and at least 40 m away from other habitats (e.g., forest, hedges). Batcorders were activated using the manufacturer’s default settings (quality 20, threshold 27 dB, posttrigger 600 ms, critical frequency 16 kHz) from 1 hr before sunset to 1 hr after sunrise. Even though acoustic sampling may underrepresent bats with low-intensity echolocation calls (e.g., *Plecotus* sp.) or be influenced by habitat elements [74,75], we conducted continuous overnight sampling using a standardized and replicated sampling scheme [76, 77], which is widely accepted for assessing bat assemblages.

For analyzing bat calls, we used the software bcAdmin v. 2.12, batIdent v. 1.03, and bcAnalyze v. 1.16. from ecoObs (Nuremberg, Germany). First, we imported and organized the data with bcAdmin. Then, we ran batIdent to automatically identify bat species using classification tree algorithms. Finally, we manually validated the automated identification with bcAnalyze given the concerns raised about the reliability of various automated identification programs [78,79]. We used the guidelines by Hammer and Zahn (2009) [80], Dietz et al. (2014) [81], and Skiba (2003) [82] for verifying dubious identifications.

The automatic classification was effective at distinguishing the calls of certain species, e.g., *Nyctalus noctula*, *Pipistrellus pipistrellus,* and *Pipistrellus pygmaeus*. In contrast, other calls were harder to distinguish, indicated by a low confidence score due to the similarity in call characteristics, e.g., *Eptesicus nilsonii*, *Nyctalus leislerii*, and *Vespertilio murinus*. In these cases, call descriptors (e.g., call frequency and the number of sequences) as well as geographic, altitudinal, and habitat preferences of the suggested species were considered to confirm the species identification. Potential misidentification could lead to a possible underestimation or overestimation of species richness. Finally, the calls of some species were not possible to distinguish due to the similarity of their calls. Hence, those species were grouped and identified as species pairs. That was the case for *Pipistrellus kuhlii* and *Pipistrellus nathusii* (grouped as Pmid), *Plecotus auritus,* and *Plecotus austriacus* (grouped as *Plecotus*), *Myotis mystacinus* and *Myotis brandtii* (grouped as Mbart), and *M. myotis* and *Myotis oxygnathus* (grouped as Mmyo). For simplicity, actual species and grouped species are referred to as “species” hereafter. We used the call sequence length (accumulated length of all sequences in seconds over one night) as a measure of bat activity [83,84]. A call sequence refers to all calls of a single pass (the devices recorded each call sequence of a passing bat and stored it in memory as an individual file). The call sequence length and the number of pulses per call sequence were highly correlated (r = 0.96). Therefore, either metric provides comparable information about bat activity. Please note that we referred to bat activity in general without distinguishing between feeding and hunting activity. We defined total species richness as the sum of different bat species per sampled pasture per night. It is important to note that passive acoustic surveys cannot provide the number of individuals of each species present.

Besides overall bat activity, we evaluated the activity of small, frequent, and infrequent species to determine whether these species were affected differently by grazing sheep. Small aerial insectivore species included *P. pipistrellus, P. pygmaeus, P. kuhlii/nathusii,* and *Hypsugo savii,* and were considered because, as aerial hawking species, they maneuver to capture flying insects in open air and along forest edges [85]*.* Frequent bat species were defined as species that occurred at ≥ 30% of the sampled pastures (S1 Table), whereas infrequent species occurred at < 30% of the sampled pastures (S1 Table). Additionally, we analyzed two bat species with high activity to assess potential species-specific responses: *N. noctula* (the largest western and central European bat and open-space forager) and *P. pygmaeus* (a small bat and an edge-space forager).

### Statistical analysis

We assessed the relationship between minimum night temperature and each response variable using Pearson correlation coefficients. As all correlations were negligible (r < 0.05), minimum night temperature was excluded from subsequent analyses. To evaluate bat activity (overall, small, frequent, infrequent species, and *N. noctula* and *P. pygmaeus*) between the three grazing categories, we performed linear mixed models (LMM; R package “glmmTMB”, [86]). Bat activity was log_10_-transformed (x + 1), grazing category was the fixed effect, and both pasture (1| pasture) and sampling date (1|date) were initially included as random factors to account for repeated measures and potential temporal variation, respectively. However, model comparisons using the Akaike Information Criterion (AIC) indicated that including date as a random effect did not improve model fit. Therefore, only pasture was retained as a random factor in the final models. We used Gaussian distribution when the response was approximately normally distributed and Tweedie distribution (for small and infrequent species, and *N. noctula*) when the response was skewed or the proportion of zeros was greater than expected by chance [87]. For analyzing differences regarding total species richness, the number of frequent and infrequent species, we performed generalized linear mixed models (GLMM, package “glmmTMB”, [86]). Similarly to the LMMs, pasture (1| pasture) was used solely as a random factor. Model assumptions for both LMMs and GLMMs were evaluated using the R package “DHARMa” [88]. To determine whether the spatial distribution of pastures influenced bat activity or species richness, we assessed spatial autocorrelation in the model residuals using Moran’s I from the “DHARMa” package [88]. The tests for spatial autocorrelation were non-significant for all models (p > 0.05), indicating no evidence of residual spatial structure. Consequently, spatial effects were not included in the final models. In cases where Poisson GLMM indicated underdispersion, we used the Conway-Maxwell-Poisson-Distribution. We conducted post-hoc pairwise comparisons using the estimated marginal means (EMMs) using the R package “emmeans” [89]. Pairwise contrasts were adjusted for multiple comparisons using the Tukey method. For interpretability, the EMMs and their confidence intervals were back-transformed to the original scale. Responses of the models were visualized with the R package “ggeffects” [90]. All statistical analyses were done in R v. 4.4.2. [91], with a significance level of 0.05.

## Results

**Fig 2:**
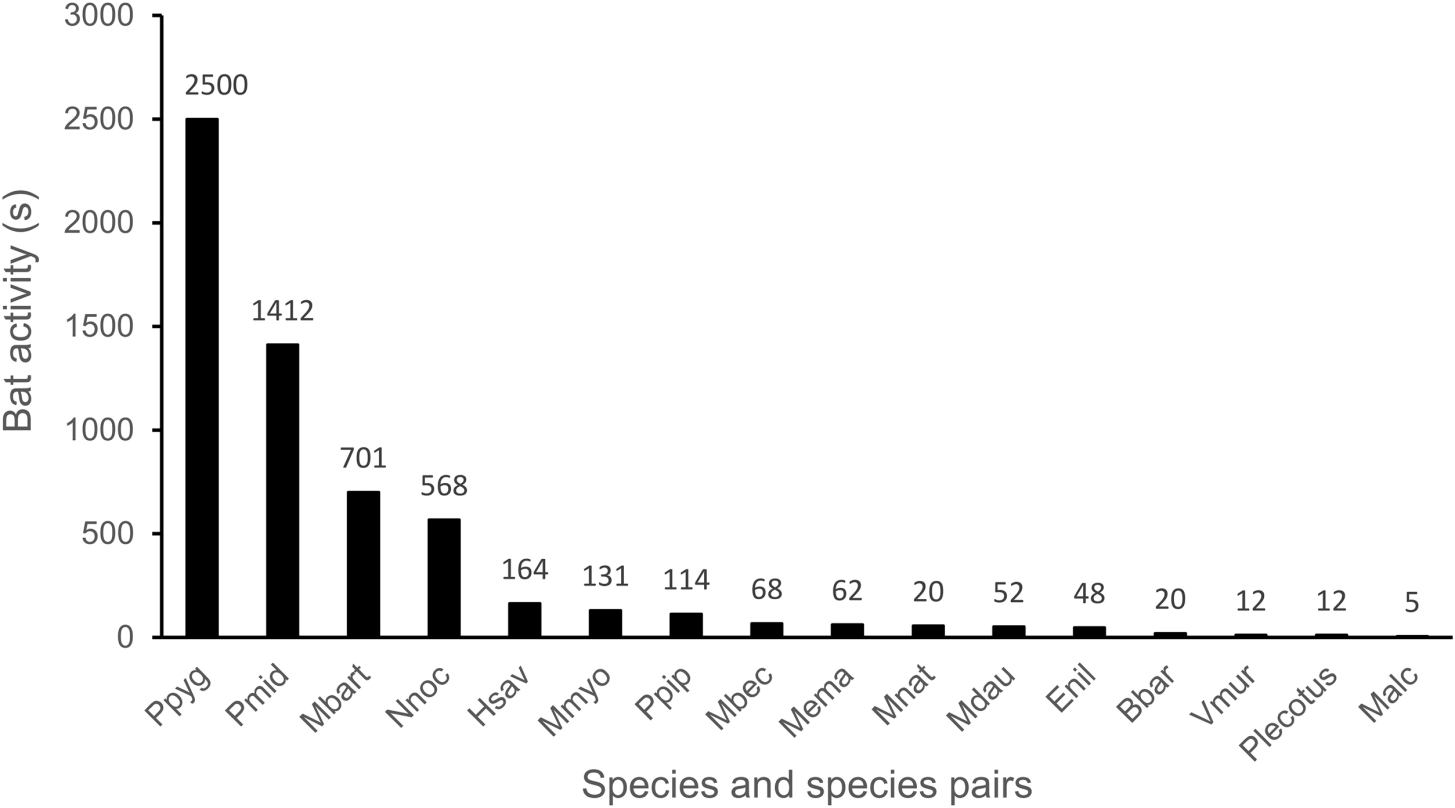
Accumulated bat activity (i.e., sequence length in seconds) for each species/species pair across all 147 sampled nights in pastures of southern Burgenland, Austria. For acronyms, please refer to S1 Table.

There were in total 8 857 s of bat activity (i.e., call sequence length), in which the highest activity was found for *P. pygmaeus* with 2 500 s, followed by the species pairs *P. kuhlii/nathusii* with 1 412 s, *M. mystacinus/brandtii* with 701 s, and *N. noctula* with 568 s (Fig 2). All other verified bat species showed less than 200 s each.

**Fig 3:**
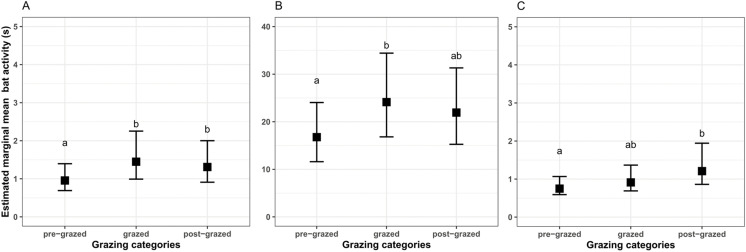
Predicted activity (accumulated sequence length per recording night) for (A) small species, (B) frequent species, and (C) *Nyctalus noctula* on sampled pastures in southern Burgenland, Austria. Mean estimates and 95% confidence intervals were based on the estimated marginal means (EMMs) back-transformed to the original scale. Letters indicate a significant pairwise comparison (*p* < 0.05). Note the different y-axis scale in panel **B.**

Overall bat activity did not differ significantly among the three grazing categories (S2 Table). The same pattern was found for the activity of infrequent species. In contrast, the activity of small species and of frequent species differed significantly among the three grazing categories (Table 1). For small species, activity was significantly higher in grazed pastures compared to pre-grazed ones (Fig 3A). Post-grazed pastures also had higher activity compared to pre-grazed, but there was no significant difference in activity between grazed and post-grazed pastures (Table 1; Fig 3A). For frequent species, activity was significantly higher in grazed pastures compared to pre-grazed (Fig 3B), but activity did not differ between pre-grazed and post-grazed, nor between grazed and post-grazed pastures (Table 1).

**Table 1:** Pairwise contrasts based on estimated marginal means (back-transformed) for overall bat activity, small species, frequent species, infrequent species, *Nyctalus noctula,* and *Pipistrellus pygmaeus* across grazing categories in southern Burgenland, Austria.

Pairwise contrast	Estimate	Std. Error	t-value	P-value
**Overall bat activity**				
Pre-grazed – grazed	−0.11	0.055	−1.976	0.122
Pre-grazed – post-grazed	−0.09	0.055	−1.616	0.242
Grazed – post-grazed	0.01	0.055	0.360	0.931
**Small species**				
Pre-grazed – grazed	4.76	0.951	−3.040	**0.007**
Pre-grazed – post-grazed	5.50	1.190	−2.366	**0.047**
Grazed – post-grazed	12.35	3.860	0.714	0.755
**Frequent species**				
Pre-grazed – grazed	−0.15	0.629	−2.394	**0.047**
Pre-grazed – post-grazed	−0.11	0.629	−1.761	0.187
Grazed – post-grazed	0.04	0.629	0.633	0.802
**Infrequent species**				
Pre-grazed – grazed	9.20	6.17	−0.122	0.992
Pre-grazed – post-grazed	4.12	1.69	−1.679	0.213
Grazed – post-grazed	4.35	1.84	−1.558	0.264
***Nyctalus noctula***				
Pre-grazed – grazed	2.19	0.354	−1.183	0.463
Pre-grazed – post-grazed	1.82	0.214	−2.611	**0.025**
Grazed – post-grazed	2.15	0.304	−1.455	0.313
***Pipistrellus pygmaeus***				
Pre-grazed – grazed	−0.11	0.069	−1.590	0.253
Pre-grazed – post-grazed	−0.09	0.070	−1.342	0.375
Grazed – post-grazed	0.02	0.069	0.248	0.967

Significant P-values are in bold.

At the species-specific level, *N. noctula* had the highest activity in post-grazed pastures compared to pre-grazed pastures (Fig 3C), whereas activity levels did not differ significantly between pre-grazed and grazed, as well as grazed and post-grazed pastures (Table 1). In contrast, LMM results did not reveal any significant effects of the grazing category on the activity of *P. pygmaeus* (S2 Table).

**Fig 4:**
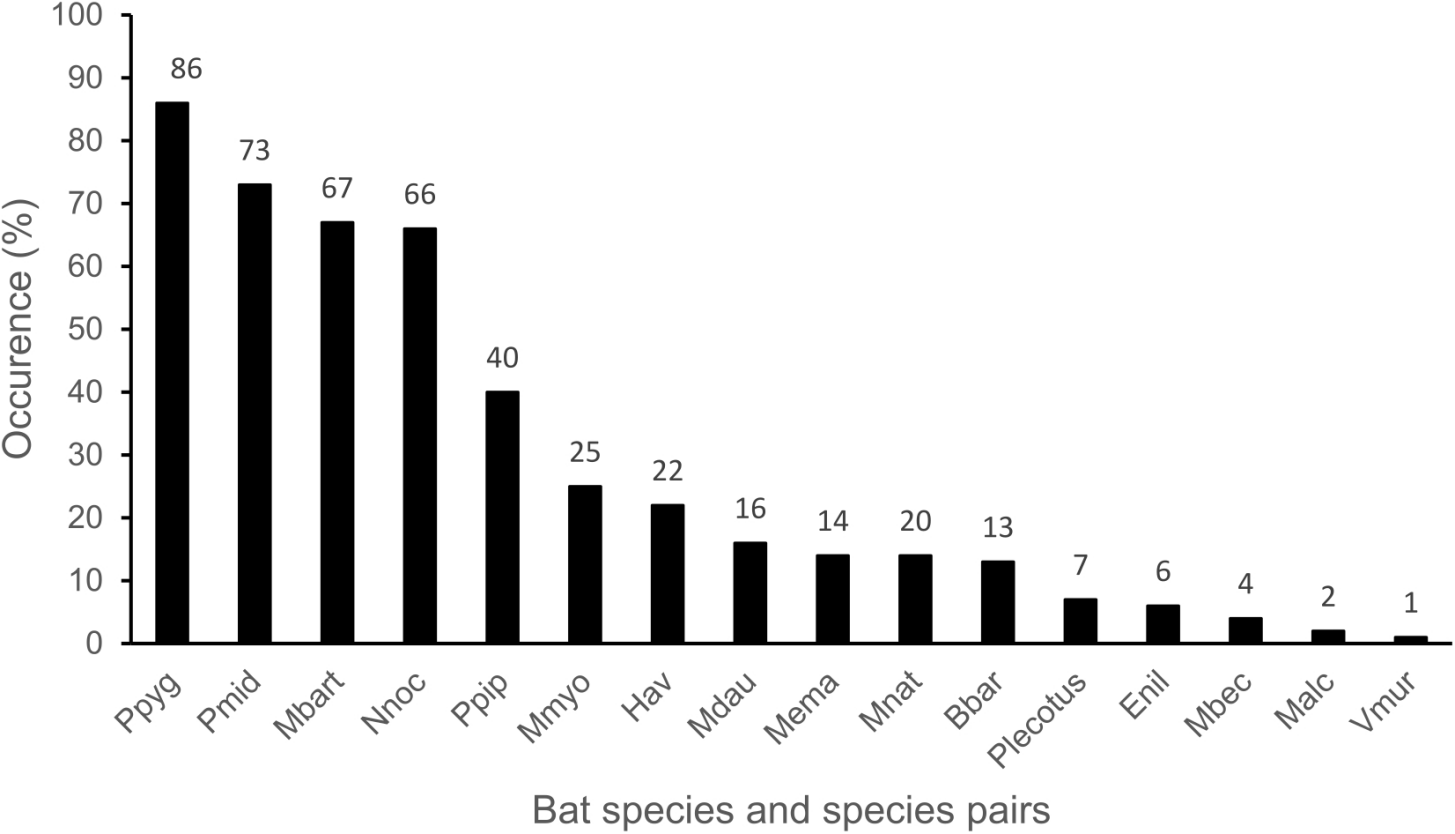
Occurrence of bat species and species pairs across all sampled pastures in southern Burgenland, Austria. Values indicate the percentage of sampled nights on which each species or species pair was detected at least once during the sampling period. For acronyms, please refer to S1 Table.

Regarding number of species, 12 species were determined at a species level and eight as species pairs (i.e., *P. kuhlii/nathusii*, *Plecotus auritus/austriacus*, *M. mystacinus/brandtii*, and *M. myotis/oxygnathus*)*.* The most predominant bat species was *P. pygmaeus,* which occurred in 86% of all sampled nights, followed by the species pairs *P. kuhlii/nathusii* with 73% and *M.* *mystacinus/brandtii* with 67%. *Nyctalus noctula* was detected in 66% and *P. pipistrellus* in 40% of the sampled nights (Fig 4). There were no significant differences among the three grazing categories regarding total species richness, number of frequent species, or number of infrequent species (Table 2). Although for total species richness and number of infrequent species, post-grazed vs. pre-grazed were nearly significant (p = 0.051 and 0.053, respectively), no individual pairwise differences were statistically significant after adjustment for multiple comparisons.

**Table 2:** Model results for total species richness, number of frequent species, and number of infrequent species on sampled pastures in southern Burgenland, Austria.

Effect type	Parameter	Estimate	Std. Error	z-value	P-value
**Total species richness**					
Conditional	(Intercept)	4.347	0.228	19.052	<0.001
	grazed	0.122	0.293	0.418	0.676
	post-grazed	0.571	0.293	1.952	0.051
Random	variance (pasture)	0.451			
	variance (residual)	2.100			
**Number of frequent species**					
Conditional	(Intercept)	1.178	0.047	24.754	<0.001
	grazed	0.018	0.063	0.288	0.773
	post-grazed	0.036	0.062	0.584	0.560
Zero-inflation	(Intercept)	−22.611	11610	−0.002	0.998
	grazed	0.227	15564	0.000	0.999
	post-grazed	0.227	15564	0.000	0.999
Random	variance (pasture)	0.012			
**Number of infrequent species**					
Conditional	(Intercept)	0.078	0.141	0.552	0.581
	grazed	0.055	0.192	0.287	0.774
	post-grazed	0.347	0.179	1.935	0.053
Random	variance (pasture)	0.002			

We report fixed-effect estimates and random-effect variance components (pasture as a random factor). Models were fitted as generalized linear mixed models (GLMMs). For frequent species, a zero-inflated GLMM was used to account for excess zeros; the zero-inflation component is reported alongside the mean-model parameters.

## Discussion

The study evaluated the influence of grazing sheep on bat activity and bat species richness by examining pastures under three grazing phases (i.e., pre-grazing, grazing, and post-grazing) in southern Burgenland, Austria. The activity of small aerial insectivores was significantly higher in both grazed and post-grazed pastures compared to pre-grazed pastures, with no significant difference observed between grazed and post-grazed pastures. Frequent species showed greater activity in grazed pastures compared to pre-grazed, but no significant differences were found between pre-grazed and post-grazed pastures or between grazed and post-grazed pastures. At the species level, *N. noctula* activity was significantly higher in post-grazed pastures compared to pre-grazed pastures. In contrast, overall bat activity, activity of infrequent species, and bat species richness (total, frequent, and infrequent species) did not differ among the three grazing phases (i.e., pre-grazed, grazed, and post-grazed).

The significantly higher activity of small aerial insectivores in our grazed and post-grazed pastures compared to pre-grazed pastures may be attributed to changes in habitat structure and prey availability created by grazing. Grazing by sheep reduces vegetation height and density, creating open spaces that are favorable for small, aerial-hawking species, such as *Pipistrellus* spp., that typically hunt for insects in open areas [92]. Additionally, the presence of livestock during grazing attracts a variety of insects, including biting midges and mosquitoes, which are preferred prey for many small aerial insectivores [51,93]. These conditions likely explain the increased activity of small aerial insectivores in grazed pastures. Previous studies have also found significantly higher activity of small bat species in pastures grazed with cattle compared to those without cattle [49], indicating that livestock presence enhances foraging opportunities for these bats [49,51].

Our finding that small aerial insectivores showed similar activity levels between grazed and post-grazed sheep pastures contrasts with previous studies using cattle, where higher activity was observed between actively grazed compared to post-grazed pastures [49,51]. This divergence suggests that livestock type influences grazing effects on bat communities. Cattle, being much larger than sheep, create greater physical disturbance and more substantial trampling effects, which can influence habitat structure. The body mass of livestock is often standardized using the livestock unit (GVE) [94], where one GVE corresponds to the mean weight of one two-year-old cow. In comparison, one adult sheep is equivalent to 0.15 GVE [95]. In our study, the sampled flock of 70 sheep equaled 10.5 GVE, or the equivalent of 10.5 cows. By contrast, the cattle flocks studied by Downs and Sanderson (2010) [51] and Ancillotto et al. (2017) [49] ranged from 20 to 60 cows (GVE) per flock, representing a much larger biomass attracting insects and a greater potential for physical impact on habitat structure. This substantial difference in livestock size and weight could explain the more pronounced effects observed in grazed vs. post-grazed pastures with cattle compared to the relatively subtle effects seen in our study with sheep.

Another interconnected factor that may explain why sheep grazing produces different patterns of activity of small aerial insectivores compared to cattle grazing is the average duration of sheep grazing on pastures, particularly in combination with our narrow and small pastures (mean size: 0.4 ha). In our study, the sheep graze each pasture for an average of one week before rotation, three times per year, resulting in brief, concentrated grazing periods. By contrast, most well-documented grazing projects with cattle employ year-round or seasonal grazing on large pastures (> 50 ha [e.g., 49,96]) as nature conservation tools [97–99], leading to more continuous grazing [100]. Thus, differences between cattle-based findings and our sheep-grazing contexts may reflect variations in grazing schedule (duration and frequency), animal numbers per unit area (stocking rate), pasture size, and management practices as much as livestock type.

Additionally, differences in the characteristics of cattle and sheep dung (size, moisture content, and decomposition rate) [101] may influence the duration and intensity of insect attraction during post-grazing periods. Adult cattle can excrete up to 25 kg of fresh dung per day [102], whereas sheep generally produce smaller quantities of dung, which are more widely distributed and with higher nutrient concentrations [103]. The wetter, bulkier cattle dung generally decomposes more quickly than the drier, pellet-like sheep dung [101,104]. These differences in moisture content and decomposition rates affect how long dung persists on pastures and, therefore, how long it acts as an attractant for insects, potentially explaining the similar activity levels observed between grazed and post-grazed sheep pastures. However, these physical differences in dung characteristics are likely compounded by the widespread use of anti-helminthics in sheep systems. Sheep require more frequent anti-helminthic treatments than cattle due to their higher vulnerability to endoparasites [105], even in organically managed farms, as was the case in our study area (personal communication Alexander Elpons, farmer). These treatments eliminate not only parasitic worms but also reduce the number of dung beetles and other dung-dwelling insects [81,96,98,106], potentially affecting bats that feed on these prey items [107]. Depending on the medication used, the prolonged excretion of anti-helminthics can persist for over 100 days [108], which could explain the similar bat activity between grazed and post-grazed pastures that we observed, as chemical effects would remain regardless of sheep presence. Differences in dung dynamics and potential anti-helminthic treatments could modulate insect pulses and bat activity. Therefore, future research should directly assess how different anti-helminthic medications (and dosages) affect dung-dwelling insect communities and, consequently, bat foraging behavior in grazing systems.

Frequent species were also more active in grazed pastures than in pre-grazed pastures, but their activity levels were similar between pre-grazed and post-grazed and between grazed and post-grazed pastures. In pre-grazed pastures, taller and denser vegetation can limit near-ground foraging and reduce access to insect prey for species that hunt within clutter [92,109,110]. By contrast, grazed pastures may provide enhanced insect prey availability due to livestock disturbance [49], creating foraging conditions that frequent species can effectively exploit. In post-grazed pastures, frequent species may still be active, as they are flexible foragers that can adjust to the intermediate resource availability present after the grazing period has ended.

We did not find differences in species richness among grazing categories, unlike other studies [e.g., 9]. However, we detected nearly all known species for Burgenland (20 out of 24) [111]. A possible explanation for the similar species richness across the three grazing categories is that our sampling did not begin at baseline conditions, i.e., before grazing implementation. Our sampled pastures have been continuously grazed for 17 years, whereas studies that have shown grazing effects on species richness [52,96,112,113] monitored pastures from the initiation of grazing. Thus, we might not have detected changes in the number of species that might have accompanied the progression of the grazing process, but rather documented the species that are currently occurring in these long-term grazed pastures. Another reason might be landscape diversity. The pastures in our study area included wet meadows, vineyards, former orchards, and dry grasslands, and are near a nature park, potentially attracting many bat species. It has been shown that landscape diversity alone positively influences species richness [49], although both landscape diversity and the presence of free-ranging cattle have explained high bat activity. In our study area, the landscape diversity, combined with the pasture management, may explain the similar species richness and relatively constant overall bat activity among pastures. While we qualitatively describe the landscape diversity, future studies should incorporate metrics such as habitat heterogeneity indices to understand their role in sheep grazing systems.

Another potential explanation for the lack of species richness differences among grazing categories is the proximity between our sampled pastures. Many of our study sites were located within relatively short distances of each other (mean distance 633.4 m), which may have facilitated bat movement and similar species assemblages across different grazing treatments. The high mobility of bats, which can travel several kilometers to foraging areas [81] and utilize multiple habitat patches within their home ranges [114], allows them to exploit resources across multiple pastures within a single foraging trip. While we found no evidence of spatial autocorrelation in our data, trees and hedgerows between pastures may have contributed to similar species occurring across all grazing categories.

Of the species analyzed, only *N. noctula* had a significantly higher activity in post-grazed pastures. *Nyctalus noctula* primarily hunts flying insects, such as moths and dipterans in the open air [115]. Following grazing, dung deposits trigger short-term increases in coprophilous insects—especially flies (Diptera)—which typically peak in the days after deposition [116]. Our post-grazing recordings (mean ≈ 4 days after sheep left the pasture) coincide with this early peak. It has been shown that *N. noctula* consistently forages in specific sites as a strategy to respond to spatial variation in ephemeral prey patches [117]. This behavioral flexibility, including the reliance on long-range, low-frequency echolocation, may explain its high activity in post-grazed pastures, where it can exploit these resource pulses [118] while avoiding potential interference from the physical presence of sheep during active grazing.

In conclusion, our study shows that in a long-established, small-pasture sheep system, small aerial insectivores exhibit higher activity in both grazed and post-grazed pastures compared to pre-grazed conditions. Frequent species were more active in grazed than pre-grazed pastures, and *N. noctula* displayed higher activity in post-grazed pastures, while species richness did not differ among grazing phases. Our findings primarily reflect immediate, short-term changes during active grazing—reduced vegetation height and trampling, elevated insect activity around livestock, and initial dung deposition— and brief post-grazing resource pulses at the temporal scale we sampled (≈4 days post-grazing) within a landscape shaped by 17 years of grazing and high landscape diversity. Comparisons with cattle-based studies should be interpreted cautiously: many cattle systems are evaluated over longer windows (weeks to months) in larger pastures with higher biomass and different dung dynamics, which can yield pronounced distinctions between grazed and post-grazed phases. Thus, under sheep grazing on small pastures and short timeframes, bats—especially small aerial insectivores and *N. noctula*—show periods of high activity in grazed and post-grazed pastures. Cross-system differences are, therefore, likely influenced as much by timeframe, pasture size, and stocking rate as by livestock type. These differences should be considered when evaluating pastoral impacts on bat communities and designing conservation-oriented grazing strategies.

## Supporting information

S1 Table List of all detected bat species during the sampling period in southern Burgenland, Austria. Acronyms and the occurrence frequency: frequent species (occurred at ≥ 30% of the sampled pastures) and infrequent species (occurred at < 30% of the sampled pastures) are included.
(DOCX)

S2 Table Model results of bat activity for all species, small species, frequent species, infrequent species, *Nyctalus noctula,* and *Pipistrellus pygmaeus* on sampled pastures in southern Burgenland, Austria. Bat activity was defined as the accumulated sequence length per recording night. We report fixed-effect estimates and random-effect variance components (pasture as a random intercept). For small species, we fitted a zero-inflated Tweedie GLMM to accommodate zero-heavy, right-skewed responses; both mean-model parameters and the zero-inflation component are reported. Significant p-values are in bold.
(DOCX)

S3 Table Raw bat activity data across the grazing categories in southern Burgenland, Austria. (CSV)
