# Supplementary material for: Do sheep-grazed pastures support insectivorous bat activity and bat species richness?
Source: PLoS One. 2026 Jan 23;21(1):e0341356. doi: 10.1371/journal.pone.0341356 (PMC12829865; doi:10.1371/journal.pone.0341356)
Supplement: S1 Table — Acronyms and the occurrence frequency: frequent species (occurred at ≥ 30% of the sampled pastures) and infrequent species (occurred at < 30% of the sampled pastures) are included. (DOCX) [file pone.0341356.s001.docx]

| Species | Acronym | Occurrence |
| --- | --- | --- |
| *Pipistrellus pipistrellus* | Ppip | Frequent |
| *Pipistrellus pygmaeus* | Ppyg | Frequent |
| *Pipistrellus kuhlii/ Pipistrellus nathusii* | Pmid | Frequent |
| *Minopterus schreibersi* | Misch | Infrequent |
| *Hypsugo savii* | Hsav | Infrequent |
| *Plecotus auritus/ Plecotus austriacus* | Plecotus | Infrequent |
| *Barbastella barbastellus* | Bbar | Infrequent |
| *Nyctalus noctula* | Nnoc | Frequent |
| *Nyctalus leiseri* | Nlei | Infrequent |
| *Eptesicus serotinus* | Eser | Infrequent |
| *Eptesicus nilsonii* | Enil | Infrequent |
| *Vespertilio murinus* | Vmur | Infrequent |
| *Myotis dasycneme* | Mdas | Infrequent |
| *Myotis daubentonii* | Mdau | Infrequent |
| *Myotis mystacinus/ Myotis brandtii* | Mbart | Frequent |
| *Myotis bechsteinii* | Mbec | Infrequent |
| *Myotis natteri* | Mnat | Infrequent |
| *Myotis emarginatus* | Mema | Infrequent |
| *Myotis alcathoe* | Malc | Infrequent |
| *Myotis myotis / Myotis oxygnathus* | Mmyo | Infrequent |
| *Rhinolophus hipposidersos* | Rhip | Infrequent |
