# Supplementary material for: Do sheep-grazed pastures support insectivorous bat activity and bat species richness?
Source: PLoS One. 2026 Jan 23;21(1):e0341356. doi: 10.1371/journal.pone.0341356 (PMC12829865; doi:10.1371/journal.pone.0341356)
Supplement: S2 Table — Bat activity was defined as the accumulated sequence length per recording night. We report fixed-effect estimates and random-effect variance components (pasture as a random intercept). For small species, we fitted a zero-inflated Tweedie GLMM to accommodate zero-heavy, right-skewed responses; both mean-model parameters and the zero-inflation component are reported. Significant p-values are in bold. (DOCX) [file pone.0341356.s002.docx]

| **Effect type** | **Parameter** | **Estimate** | **Std. Error** | **z-value** | **P-value** |
| --- | --- | --- | --- | --- | --- |
| **Overall bat activity** | |  |  |  |  |
| Conditional | (Intercept) | 1.563 | 0.050 | 31.103 | **<0.001** |
|  | grazed | 0.109 | 0.055 | 1.976 | 0.053 |
|  | post-grazed | 0.089 | 0.055 | 1.616 | 0.106 |
| Random | variance (pasture) | 0.048 |  |  |  |
|  | variance (residual) | 0.075 |  |  |  |
| **Small species** | |  |  |  |  |
| Conditional | (Intercept) | -0.019 | 0.065 | -0.302 | 0.763 |
|  | grazed | 0.169 | 0.056 | 3.040 | **0.002** |
|  | post-grazed | 0.131 | 0.055 | 2.366 | **0.018** |
| Zero-inflation | (Intercept) | -3.887 | 1.026 | -3.787 | **<0.001** |
|  | grazed | 1.465 | 1.152 | 1.272 | 0.203 |
|  | post-grazed | 0.726 | 1.256 | 0.578 | 0.563 |
| Random | variance (pasture) | 0.123 |  |  |  |
| **Frequent species** | |  |  |  |  |
| Conditional | (Intercept) | 1.249 | 0.061 | 20.251 | **<0.001** |
|  | grazed | 0.151 | 0.063 | 2.394 | **0.017** |
|  | post-grazed | 0.111 | 0.063 | 1.761 | 0.078 |
| Random | variance (pasture) | 0.089 |  |  |  |
|  | variance (residual) | 0.096 |  |  |  |
| **Infrequent species** | |  |  |  |  |
| Conditional | (Intercept) | -0.490 | 0.098 | -4.997 | **<0.001** |
|  | grazed | 0.016 | 0.131 | 0.122 | 0.903 |
|  | post-grazed | 0.211 | 0.126 | 1.679 | 0.093 |
| Random | variance (pasture) | 0.032 |  |  |  |
| ***Nyctalus noctula*** | |  |  |  |  |
| Conditional | (Intercept) | -1.175 | 0.169 | -6.940 | **<0.001** |
|  | grazed | 0.245 | 0.207 | 1.183 | 0.237 |
|  | post-grazed | 0.514 | 0.197 | 2.611 | **0.009** |
| Random | variance (pasture) | 0.122 |  |  |  |
| ***Pipistrellus pygmaeus*** | |  |  |  |  |
| Conditional | (Intercept) | 0.712 | 0.084 | 8.433 | **<0.001** |
|  | grazed | 0.111 | 0.069 | 1.590 | 0.112 |
|  | post-grazed | 0.094 | 0.069 | 1.342 | 0.180 |
| Random | variance (pasture) | 0.230 |  |  |  |
|  | variance (residual) | 0.119 |  |  |  |
